# Isoquercetin Improves Inflammatory Response in Rats Following Ischemic Stroke

**DOI:** 10.3389/fnins.2021.555543

**Published:** 2021-02-09

**Authors:** Yunwei Shi, Xinyi Chen, Jiaxing Liu, Xingjuan Fan, Ying Jin, Jingxiao Gu, Jiale Liang, Xinmiao Liang, Caiping Wang

**Affiliations:** ^1^Key Laboratory of Neuroregeneration of Jiangsu and Ministry of Education, Co-innovation Center of Neuroregeneration, Nantong University, Nantong, China; ^2^Department of Neurology, Affiliated Hospital of Nantong University, Nantong, China; ^3^Medical School, Nantong University, Nantong, China; ^4^Dalian Institute of Chemical Physics, Chinese Academy of Sciences, Dalian, China

**Keywords:** isoquercetin, neuroprotection, ischemia, inflammation, apoptosis, stroke

## Abstract

Inflammatory response contributes to brain injury after ischemia and reperfusion (I/R). Our previous literature has shown isoquercetin plays an important role in protecting against cerebral I/R injury. The present study was conducted to further investigate the effect of isoquercetin on inflammation-induced neuronal injury in I/R rats with the involvement of cyclic adenosine monophosphate (cAMP)/protein kinase A (PKA) and inhibitor of NF-κB (I-κB)/nuclear factor-kappa B (NF-κB) signaling pathway mediated by Toll-like receptor 4 (TLR4) and C5a receptor 1 (C5aR1). *In vivo* middle cerebral artery occlusion and reperfusion (MCAO/R) rat model and *in vitro* oxygen-glucose deprivation and reperfusion (OGD/R) neuron model were used. MCAO/R induced neurological deficits, cell apoptosis, and release of cytokines such as tumor necrosis factor (TNF)-α, interleukin (IL)-1β, and IL-6 in ischemic brain in rats. Simultaneously, the expression of TLR4 and C5aR1 was significantly up-regulated in both MCAO/R rats and OGD/R neurons, accompanied with the inhibition of cAMP/PKA signaling and activation of I-κB/NF-κB signaling in the cortex of MCAO/R rats. Over-expression of C5aR1 in neurons induced decrease of cell viability, exerting similar effects with OGD/R injury. Isoquercetin acted as a neuroprotective agent against I/R brain injury to suppress inflammatory response and improve cell recovery by inhibiting TLR4 and C5aR1 expression, promoting cAMP/PKA activation, and inhibiting I-κB/NF-κB activation and Caspase 3 expression. TLR4 and C5aR1 contributed to inflammation and apoptosis via activating cAMP/PKA/I-κB/NF-κB signaling during cerebral I/R, suggesting that this signaling pathway may be a potent therapeutic target in ischemic stroke. Isoquercetin was identified as a neuroprotective agent, which maybe a promising therapeutic agent used for the treatment of ischemic stroke and related diseases.

## Introduction

Stroke remains one of the greatest causes of mortality and morbidity and imposes an immense economic burden worldwide (Donkor, 2018; Fitzmaurice et al., 2019). Despite this, there exist only two approved treatment options, tissue plasminogen activator (tPA) and endovascular thrombectomy, but short therapeutic windows and high risk of additional injury limit their applicability (Keep et al., 2014; Jiang et al., 2018; Shah et al., 2020). Ischemic stroke, which comprises 80–85% of all stroke cases, involves a reduction of blood supply that leads to oxygen and nutrient deprivation and thus cell death in the brain (Zhang et al., 2017; Deng et al., 2019). Ischemic stroke initiates complex processes including inflammation, oxidative stress, excitotoxicity, and apoptosis, therefore resulting in the neuronal death (Khoshnam et al., 2017). Middle cerebral artery occlusion (MCAO) is the most common cause of ischemic stroke in humans (Cespedes et al., 2019). Neurological deficits, possibly persisting for many months after stroke onset, are the typical examples of stroke sequelae arising from MCAO. After ischemic stroke, the ischemic region can be divided into ischemic core and the penumbra. Necrotic cell death occurs rapidly after MCAO in the core region, whereas neuronal damage develops more slowly in the penumbral area (Furlan et al., 1996; Xu et al., 2003). Among the factors contributing to neuronal delayed damage, inflammation and apoptosis play key roles in the penumbra (Xu et al., 2003). Inflammatory response and its uncovering mechanisms may provide preventative therapies against subsequent neuronal cell death in ischemia-related events (Dalli et al., 2015; Zimmer et al., 2016). The innate immune system plays a crucial role in the inflammatory responses after ischemic stroke through the activation of receptors (Yang et al., 2008). In particular, the concerted activation of two sorts of the membrane receptors of the innate immune system, TLRs and complement, results in rapid inflammatory responses after injury (Yang et al., 2008; Zaal et al., 2017a). It has been demonstrated that Toll-like receptor 4 (TLR4) and C5a receptor 1 (C5aR1) dampen the pro-inflammatory potential of human monocyte-derived dendritic cells (moDCs) (Zaal et al., 2017a,b). Moreover, more than 20 compounds by modulating C5aR1 are in preclinical development or have already reached clinical trials (Woodruff et al., 2011; Ricklin and Lambris, 2016). The fact emphasizes the significance for further elucidation of regulation of inflammation by C5aR1 and TLR4.

Isoquercetin is a dietary flavonoid present in a variety of medicinal and dietary plants, including vegetables, herbs, and flowers (Zhou et al., 2014; Michalcova et al., 2019). It has been shown to have numerous therapeutic properties, including anti-inflammatory, antioxidant, and anti-allergic activities (Kangawa et al., 2017; Fayed et al., 2019; Qiu et al., 2019). Moreover, our previous study has demonstrated that isoquercetin has neuroprotective effects after MCAO and reperfusion (MCAO/R) injury in rats (Wang et al., 2017). Although isoquercetin can protect the hippocampus after ischemic stroke by inhibiting inflammation and apoptosis shown by our previous study, the mechanisms responsible for its neuroprotective effects remain not adequately clear. In the present study, we further determined the effects of isoquercetin on the cortex injury in rats after MCAO/R and in primary culture of rat cortical neurons subjected to oxygen-glucose deprivation and reperfusion (OGD/R). In addition, the action of isoquercetin suppressing inflammation response after ischemia and reperfusion (I/R) by inhibiting the TLR4 and C5aR1 signaling pathway *in vivo* and *in vitro* was evaluated in order to further elucidate its precise neuroprotective mechanisms.

## Materials and Methods

### Chemicals and Reagents

Isoquercetin (≥ 98%, HPLC) was bought from Shanghai PureOne Biotechnology (Shanghai, China). The 1,3-[4,5-dimethyl-2-thiazolyl]-2,5-diphenyl-2-tetrazolium bromide (MTT) and 2,3,5-triphenyltetrazolium chloride (TTC) were purchased from Sigma-Aldrich (Saint Louis, MO, United States). *In situ* cell death detection kit, TMR red (Roche, Mannheim, Germany), was used for terminal deoxynucleotidyl transferase (TdT)-mediated dUTP-biotin nick end labeling (TUNEL) assay. Protein kinase A (PKA) kinase activity assay kit (ab139435) was purchased from Abcam Ltd. Tumor necrosis factor-α (TNF-α), interleukin-1β (IL-1β), and IL-6 enzyme-linked immunosorbent assay (ELISA) kits were purchased from Proteintech Group, Inc. (CA, United States). Cyclic adenosine monophosphate (cAMP) ELISA kit was bought from Elabscience Biotechnology Co., Ltd. C5aR1 RNAscope^®^ Fluorescent Multiplex Kit was purchased from Advanced Cell Diagnostics, Inc. (Hayward, CA, United States). All standard culture reagents were obtained from Gibco (Grand Island, NY, United States). For Western blotting, immunohistochemistry (IHC), and immunofluorescence analyses, the antibodies used are listed in Supplementary Table S1. Other chemical reagents were commercially available with analytical grade.

### Animals

Male Sprague–Dawley (SD) rats, weighing 160–180 g, were provided by the Experimental Animal Center of Nantong University. Animals were kept on a 12 h light and dark cycle and housed individually with access to food and water *ad libitum* (20 ± 1°C). All experimental and animal handling procedures were carried out in accordance with animal care guidelines and conforming to the Chinese law for the Protection of Animals.

### Rat Transient Focal Cerebral Ischemia Model and Isoquercetin Treatment

**FIGURE 1:**
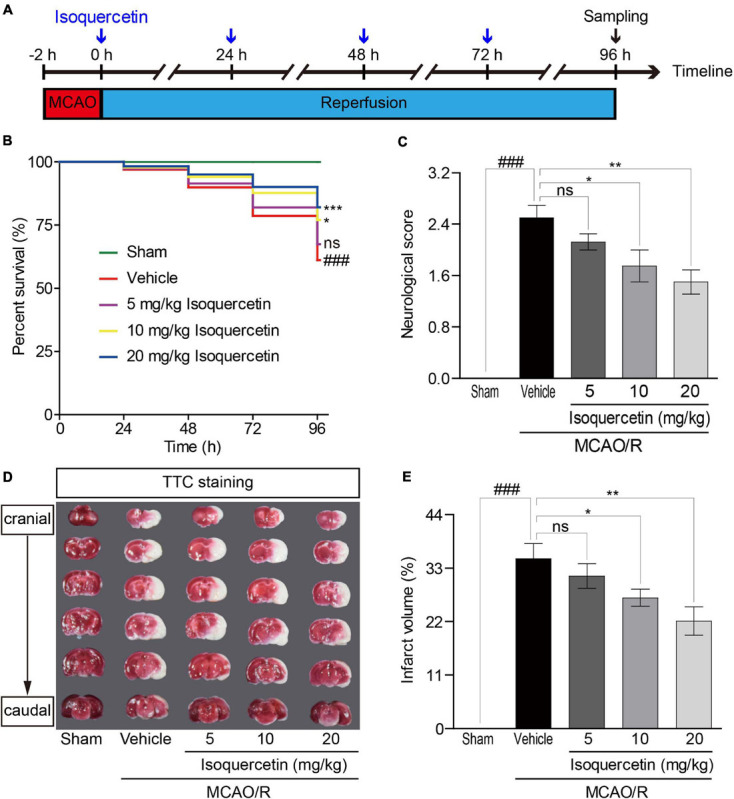
Effects of isoquercetin on brain injury in MCAO/R-induced rats. Isoquercetin (5, 10, and 20 mg/kg/day) was orally administered after 2 h MCAO for four consecutive days. **(A)** Scheme of the animal experimental protocol. **(B)** The survival percentage of rats after MCAO/R and isoquercetin treatments (*n* = 45). **(C)** Neurological deficit scores after MCAO/R and oral administration of different doses of isoquercetin (*n* = 6). **(D)** Representative coronal brain sections (2 mm-thick, measured in six serial coronal sections arranged from cranial to caudal regions) from sham-operated, vehicle-treated, or isoquercetin-treated rats stained with 2% TTC staining. Red colored regions in the TTC-stained sections are non-ischemic regions, and pale-colored regions indicate the ischemic portions of the brain. **(E)** Quantitative analyses of infarct volumes. The infarct volumes from sham groups were all equal to zero. The influence of edema on infarct volume was corrected by standard methods (volume of contralateral hemisphere – volume of non-ischemic ipsilateral hemisphere), with infarcted volume expressed as a percentage of the contralateral hemisphere (*n* = 6). Results were present in mean values ± SEM. ^###^*p* < 0.001 vs. sham group; *p < 0.05, **p < 0.01, and ***p < 0.001 vs. vehicle-treated group.

Transient MCAO/R was performed as described previously (Ortega et al., 2013; Wang et al., 2017). Rats were divided into sham-operated rats and MCAO/R rats. MCAO/R rats were subdivided into vehicle-treated rats and isoquercetin-treated rats. Two hours after MCAO, isoquercetin-treated rats were administrated with three doses at 5, 10, and 20 mg/kg by gavage once a day for four consecutive days. Rats were sacrificed at 96 h after MCAO/R injury. Isoquercetin was prepared in DMSO and diluted in saline to the final concentration (DMSO final concentration < 0.5%). In vehicle-treated group, animals received MCAO for 2 h and reperfusion with oral administration of 1 ml normal saline and 0.5% of DMSO for four consecutive days. The animal experiment was carried out according to the working procedure (Figure 1A).

### Neurological Deficit Scores and Cerebral Infarct Assessment

The neuroscore assessment was performed as described previously (Wang et al., 2017). The cerebral infarct was measured by TTC staining. In brief, before operation, rats were euthanized under deep anesthesia, and the brain was quickly dissected and cut into 2 mm-thick coronal sections using a rat brain matrix (RWD, Shenzhen, China). The brain slices were immediately placed in PBS containing 0.5% TTC at 37°C for 20 min. And then, the slices were fixed in buffered 4% formaldehyde solution for 15 min at room temperature, followed by image capture with a camera (Canon, Tokyo, Japan) and analysis of the infarct volumes by the image analysis system (Image-Pro Plus 5.1, Leica Imaging System Ltd., Cambridge, United Kingdom). The cerebral infarct volume was measured using a subtraction method to control for edema. The infarct volume controlled for edema (%) = [total infarct volume – (ipsilateral hemisphere volume – contralateral hemisphere volume)] / contralateral hemisphere volume × 100.

### Measurements of the cAMP Level and PKA Activity in Plasma and Cortical Tissue and the Levels of TNF-α, IL-1β, and IL-6 in Plasma of Rats

Plasma samples were collected after the sacrifice operation. cAMP levels; PKA activity in plasma and cortical tissue; and the levels of TNF-α, IL-1β, and IL-6 in plasma were measured with commercial kits according to the manufacturer’s instructions.

### Histopathology

**FIGURE 2:**
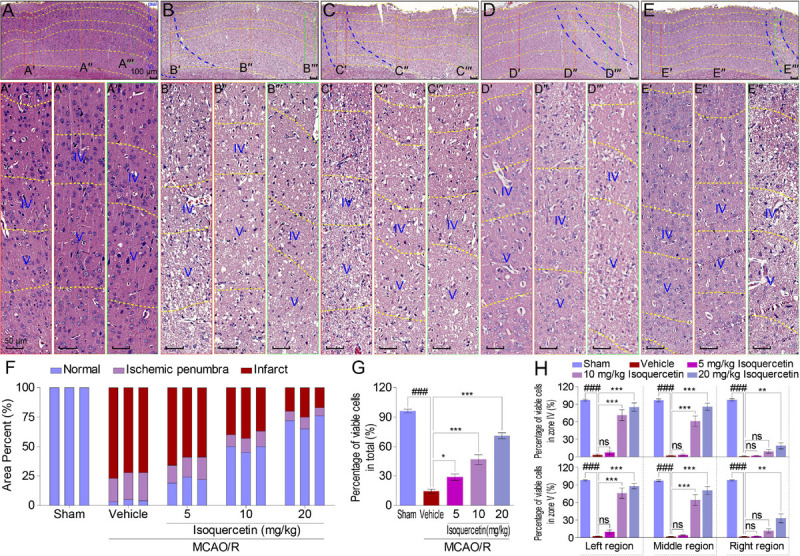
Effects of isoquercetin on cells in the parietal cortex of MCAO/R-induced rats by H&E staining. **(A–E)** Representative micrographs of the parietal cortex region in sham-operated, vehicle-treated, or isoquercetin-treated rats (**A**, sham; **B**, vehicle; **C**, 5 mg/kg isoquercetin; **D**, 10 mg/kg isoquercetin; **E**, 20 mg/kg isoquercetin). The parietal cortex was divided into seven layers (layer pial and I–VI). The penumbra region in the parietal cortex was marked with two blue dash lines. The left, middle, and right regions were marked with red-, orange-, and green-colored rectangles, and numbered as **(A′–E″′)**. **(A′–E″′)** The zoom-in micrographs of marked regions in **(A–E)**. The layers IV and V were signed for further quantitative analyses. **(F)** Quantitative analyses of the area percentage of ischemic penumbra area and infarct area in the parietal cortex of sham-operated, vehicle-treated, and isoquercetin-treated rats (*n* = 3). **(G)** Quantitative analyses of the percentage of viable cells in the parietal cortex of sham-operated, vehicle-treated, and isoquercetin-treated rats (*n* = 9). **(H)** Quantitative analyses of the percentage of viable cells within layers IV and V of the parietal cortex in sham-operated, vehicle-treated, and isoquercetin-treated rats (*n* = 3). Data were represented as mean ± SEM. ^###^*p* < 0.001 vs. sham group; *p < 0.05, **p < 0.01, and ***p < 0.001 vs. vehicle-treated group.

Brain slices, containing parietal cortex, were used for hematoxylin and eosin (H&E) assay (Wang et al., 2017). For convenience, to count the cells in stained cortex sections, the cerebral cortex was divided into seven layers (Figure 2A) according to the reported method (Cooper, 2008; Stiles and Jernigan, 2010). The total number of normal and pathological neurons in at least six different ischemic zones of the ipsilateral brain region in the parietal cortex of the tissue sections obtained from at least three animals per group was counted. The ischemic penumbra area and infarct area in the parietal cortex of rats were assayed. The average number of viable neurons in different regions was counted. The results were expressed as a percentage of viable cells (Nunez-Figueredo et al., 2014).

### TUNEL Staining

Cryostat brain sections were used in TUNEL staining. The staining procedure was according to the manufacturer’s protocol. Apoptotic (TUNEL-positive) cells were detected as localized bright red signals in a red background under a DMR fluorescence microscope (Leica Microsystems, Wetzlar, Germany). Data are expressed as the ratio of apoptotic cells to total cells.

### Immunohistochemistry Staining

At 96 h post MCAO/R, rats were anesthetized and sacrificed. Brain tissues were harvested for IHC detections of TLR4, TNF-α, IL-1β, IL-6, and Caspase 3 according to the method described previously (Wang et al., 2017). The results were assessed in a double-blinded approach and expressed according to the level of immunoreactivity. The weakest immunoreactivity was given a score of 1, whereas the highest immunoreactivity was 5.

### Immunofluorescence Staining

Brain sections were harvested for detection of TLR4 in the parietal cortex of rats after MCAO/R injury and isoquercetin treatments by immunofluorescence staining. Brain sections were immunostained at room temperature for 1 h with TLR4 antibody (1:200), followed by the appropriate fluorescent secondary antibody (1:300, Proteintech). Tissue sections were examined with a fluorescence microscope. The density of immunopositive cells was determined in the parietal cortex.

### Cell Culture

Primary cultures of rat cortical neurons were obtained from day 17 to 18 SD rat embryos. Pregnant rats were obtained from the Experimental Animal Center of Nantong University (Nantong, Jiangsu, China). Cell culture procedure was carried out as described previously (Brewer et al., 1993; Wang et al., 2017).

### OGD/R and Isoquercetin Treatment

**FIGURE 5:**
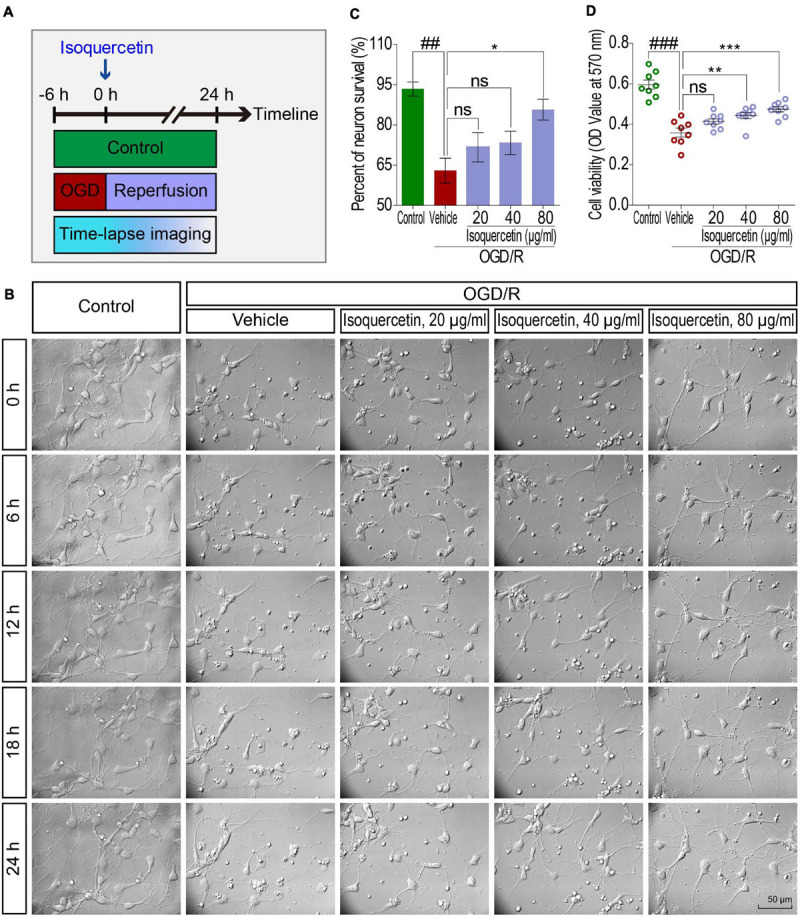
Effects of isoquercetin (20, 40, and 80 μg/ml) on primary culture of rat cortical neurons after OGD/R injury. **(A)** Scheme of the experimental protocol in primary culture of rat cortical neurons. **(B)** The photographs by time-lapse imaging at different time points in 24 h after OGD/R and isoquercetin treatment. **(C)** The percentage of neuron survival at 24 h after isoquercetin treatment (*n* = 3). **(D)** Cell viability determination with MTT assay (*n* = 8). Data were expressed as mean ± SEM. ^##^*p* < 0.01 and ^###^*p* < 0.001 vs. control group; *p < 0.05, **p < 0.01, and ***p < 0.01 vs. vehicle-treated group.

The induction of OGD/R was based on the method previously reported (Wang et al., 2017). The working doses of isoquercetin were 20, 40, and 80 μg/ml in the culture medium. Control culture plates were always maintained in an incubator with 5% CO_2_ at 37°C, without exposure to OGD/R and isoquercetin treatment. In the vehicle group, cells were exposed to 6 h OGD and 24 h reperfusion. The *in vitro* experiment was carried out according to the procedure in Figure 5A.

### Cell Viability Assay and Morphological Analysis of Cortical Neurons

Cell viability was determined by MTT assay. Time-lapse analysis was performed to monitor the morphological changes during OGD/R and isoquercetin treatment as our previously reported (Wang et al., 2017).

### Electrotransfection and Drug Treatment

Primary culture of rat cortical neurons grown on a 96-well plate was co-transfected with EGFP-N1 and C5aR1. After 24 h, the neurons were treated with 20, 40, and 80 μg/ml of isoquercetin. Cell viability and C5aR1 expression assays were detected 6 h later. Transfection was carried out by Eporator according to the directions of the manufacturer.

### xCELLigence Assay

xCELLigence assay was performed to detect the effect of isoquercetin on normal neurons as previously reported (Shi et al., 2017). Six concentrations of isoquercetin at 10, 20, 40, 80, 160, and 320 μg/ml were detected.

### Fluorescence *in situ* Hybridization

C5aR1 mRNA expressions in the parietal cortex of rats after MCAO/R and isoquercetin treatment were determined by C5aR1 RNAscope^®^ Fluorescent Multiplex Kit according to the supplier’s instruction. Positive green signals in the parietal cortex were detected under a Pannoramic MIDI Digital Slide Scanner (3DHISTECH Ltd., Budapest, HUNGARY). Data were expressed by the fluorescence intensity.

### Quantitative Real-Time PCR

mRNA levels of C5aR1 in the parietal cortex of ischemic brain of rats were evaluated by quantitative real-time PCR (qPCR). Total RNA was extracted using TRIzol (Invitrogen, Carlsbad, CA) and was reverse transcribed into cDNA using an Omniscript RT kit (Qiagen, Valencia, CA). qPCR was performed using Fast-Plus EvaGreen^®^ qPCR Master Mix kit (Biotium, Hayward, CA). The relative expression level for TLR4 and C5aR1 was normalized to the housekeeping gene GAPDH. Each sample was tested in triplicate, and the 2^–ΔΔCT^ method was used to calculate the relative transcription data (Rodrigues Hell et al., 2009). The following primers were used: *C5aR1_fwd* TACCACAGAACCCAGGAGGA, *C5aR1_rev* CGCTTCGGGAGGTGAATG, *GAPDH_fwd* TGAG GCCGGTGCTGAGTATGT, and *GAPDH_rev* CAGTCTTCTG GGTGGCAGTGAT.

### Western Blot Analysis

Total protein of the parietal cortex was extracted, and the protein samples were subjected to protein quantification with a BCA protein assay kit. Western blot analysis of TNF-α, IL-1β, IL-6, Caspase 3, TLR4, PKA, p-PKA, nuclear factor-kappa B (NF-κB), p-NF-κB, inhibitor of NF-κB (I-κB), and p-I-κB was performed as previously described (Wang et al., 2017).

### Statistical Analysis

GraphPad Prism 5 software (GraphPad Software Inc., United States) was used for statistical analysis. Data were expressed as mean ± SEM. Comparisons among different groups were performed using a one-way analysis of variance (ANOVA) followed by post-test. Prior to ANOVA analysis, Shapiro–Wilk test was used to verify whether the data fulfilled Gaussian distribution. The Kaplan–Meier estimator/test was used to produce the survival curves. Differences were considered statistically significant at *p* < 0.05.

## Results

### Isoquercetin Alleviated Brain Injury Induced by MCAO/R

To evaluate the impact of isoquercetin on the brain injury induced by ischemic stroke, we applied MCAO/R-induced rat model and isoquercetin administration procedure as described in Figure 1A. As shown in Figure 1B, isoquercetin treatment significantly enhanced the survival percentage of MCAO/R-injured rats. Also, isoquercetin treatment significantly decreased neurological score in rats after MCAO/R injury (Figure 1C). We further detected the effects of isoquercetin treatment on the infarct volume in brain by TTC staining (Figures 1D,E). Isoquercetin treatment significantly decreased the infarct volume in MCAO/R-injured rats.

Moreover, we examined the effect of isoquercetin on the cortical cells in sham-operated, vehicle-treated, or isoquercetin-treated rats by H&E staining (Figure 2). Isoquercetin treatment apparently reduced the ischemic region displayed by penumbra site signed with blue dash lines (Figures 2B–E). Comparing with MCAO/R-injured rats, the penumbra and infarct areas were obviously decreased by isoquercetin treatment (Figure 2F), and the percentage of viable cells were significantly increased in isoquercetin-treated rats (Figure 2G). The areas of the left, middle, and right zone in the similar location of the parietal cortex slices in different groups were selected to illustrate the cell status. These areas were, respectively, framed with red, orange, or green rectangular boxes in every group. The selected zones were zoomed in and divided into different layers with yellow dashed lines (Figures 2A′–E″′). Layers IV and V are fully displayed in the enlarged images of different groups. Thus, we counted the viable cells in these two equivalent regions of the parietal cortex in rats. In layers IV and V of the parietal cortex, isoquercetin treatment significantly enhanced the percentage of viable cells, especially at the dosages of 10 and 20 mg/kg (Figure 2H).

### Isoquercetin Alleviated MCAO/R-Induced Inflammatory Responses

**FIGURE 3:**
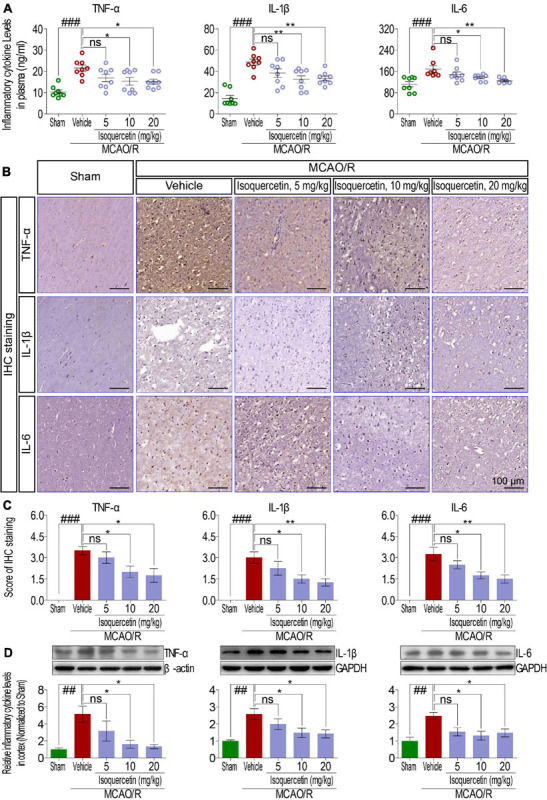
Effects of isoquercetin on three inflammatory cytokines, TNF-α, IL-1β, and IL-6, in the plasma and the cortex of sham-operated, vehicle-treated, and isoquercetin-treated rats. **(A)** TNF-α, IL-1β, and IL-6 levels in the plasma of rats (*n* = 8). **(B)** Representative micrographs of IHC staining of TNF-α, IL-1β, and IL-6 in the parietal cortex of sham-operated, vehicle-treated, and isoquercetin-treated rats. **(C)** Assessment analyses of IHC score of TNF-α, IL-1β, and IL-6 in the parietal cortex of sham-operated, vehicle-treated, and isoquercetin-treated rats (*n* = 4). **(D)** Western blot analyses for the relative protein levels of TNF-α, IL-1β, and IL-6 in the parietal cortex of sham-operated, vehicle-treated, and isoquercetin-treated rats (*n* = 3). Data were expressed as mean ± SEM. ^##^*p* < 0.01 and ^###^*p* < 0.001 vs. sham group; *p < 0.05 and **p < 0.01 vs. vehicle-treated group.

As shown in Figure 3, we examined the effects of isoquercetin on the levels of TNF-α, IL-1β, and IL-6 in the plasma and parietal cortex of sham-operated, vehicle-treated, or isoquercetin-treated rats. ELISA assay demonstrated that isoquercetin at doses of 10 and 20 mg/kg decreased the levels of TNF-α, IL-1β, and IL-6 in the plasma (Figure 3A). The effects of isoquercetin on the expression of TNF-α, IL-1β, and IL-6 in the parietal cortex of rats after MCAO/R injury were also detected by IHC staining (Figures 3B,C) and western blot (Figure 3D). The results further demonstrated that isoquercetin at doses of 10 and 20 mg/kg inhibited the expression levels of TNF-α, IL-1β, and IL-6 in the parietal cortex of MCAO/R-exposed rats. There were no significant differences in the levels of TNF-α, IL-1β, and IL-6 in the plasma and brain of rats between the vehicle and 5 mg/kg isoquercetin-treated groups.

### Isoquercetin Alleviated MCAO/R-Induced Cell Apoptosis

**FIGURE 4:**
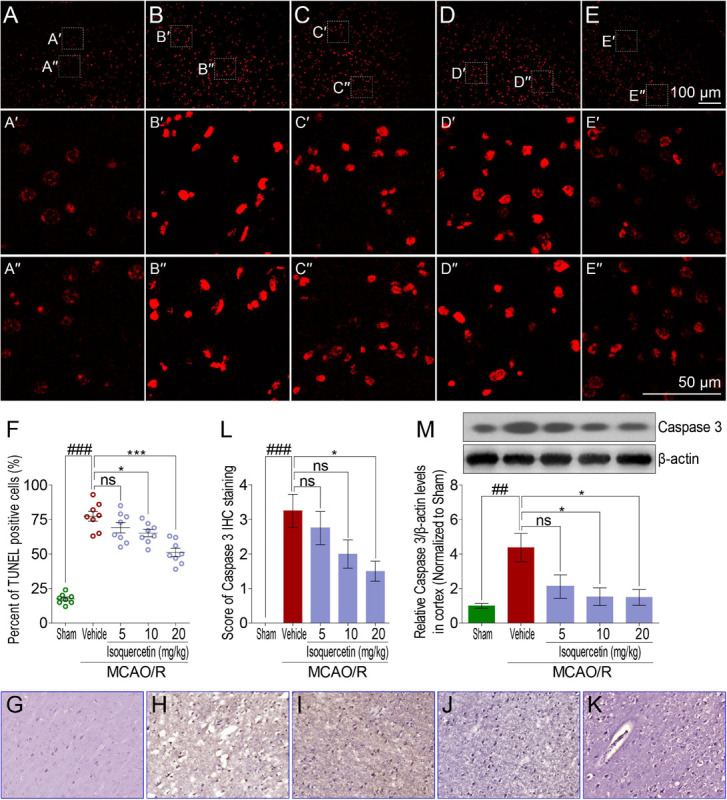
Effects of isoquercetin on cell apoptosis in the parietal cortex of sham-operated, vehicle-treated, and isoquercetin-treated rats. **(A–E)** Representative micrographs of cell apoptosis analyses of the parietal cortex by TUNEL staining (**A**, sham; **B**, vehicle; **C**, 5 mg/kg isoquercetin; **D**, 10 mg/kg isoquercetin; **E**, 20 mg/kg isoquercetin). **(A′–E″)** The zoom-in micrographs of marked regions in **(A–E)**. **(F)** Quantitative analyses of apoptotic cells (featured by bright red signals) in cell population (*n* = 8). **(G–K)** Representative micrographs of IHC staining of Caspase 3 in the parietal cortex of sham-operated, vehicle-treated, and isoquercetin-treated rats (**G**, sham; **H**, vehicle; **I**, 5 mg/kg isoquercetin; **J**, 10 mg/kg isoquercetin; **K**, 20 mg/kg isoquercetin). **(L)** Assessment analyses of IHC score of Caspase 3 in the parietal cortex of sham-operated, vehicle-treated, and isoquercetin-treated rats (*n* = 4). **(M)** The protein level of Caspase 3 in the parietal cortex of rats were assayed by western blot analysis after MCAO/R injury and isoquercetin treatment. Data were expressed as mean ± SEM (*n* = 3). ^##^*p* < 0.01 and ^###^*p* < 0.001 vs. sham group; *p < 0.05 and ***p < 0.001 vs. vehicle-treated group.

Comparing with sham group, TUNEL assay showed that MCAO/R injury induced obvious elevation of apoptosis cells in the parietal cortex of rats (Figures 4A,B). Isoquercetin at doses of 10 and 20 mg/kg significantly decreased the numbers of apoptotic cells in the parietal cortex (Figures 4C–F). We further examined the effect of isoquercetin on the expression level of Caspase 3 in the parietal cortex of MCAO/R-induced rats by IHC staining and western blot (Figures 4G–L,M). The results demonstrated that isoquercetin treatment obviously inhibited the expression level of Caspase 3 in the parietal cortex of MCAO/R-exposed rats (Figures 4L,M).

### Isoquercetin Enhanced Neuron Survival After OGD/R Injury

We further detected the effect of isoquercetin treatment in primary culture of cortical neurons by OGD/R model. By time-lapse imaging control of vehicle- and isoquercetin-treated cortical neurons during 24 h after OGD injury (Figure 5B and Supplementary Movies 1–5), we found that there was obvious enhancement in neuron viability of isoquercetin-treated neurons. It was also observed that the percentage of neuron survival was significantly increased in 80 μg/ml isoquercetin-treated group after OGD/R exposure (Figure 5C). Cell viability was also significantly increased in 40 and 80 μg/ml isoquercetin-treated groups after OGD/R exposure (Figure 5D).

To visualize the side effect of isoquercetin on normal cortical neuron, we conducted a time-lapse monitoring on the isoquercetin-treated neurons with RTCA-MP xCELLigence system. After 24 h isoquercetin treatment at six different doses (10, 20, 40, 80, 160, and 320 μg/ml) on normal cortical neurons, no significant toxicity was found below 160 μg/ml (Supplementary Figure S1).

### Isoquercetin Elicited Neuroprotective Effects by Mediating TLR4 Signaling

**FIGURE 6:**
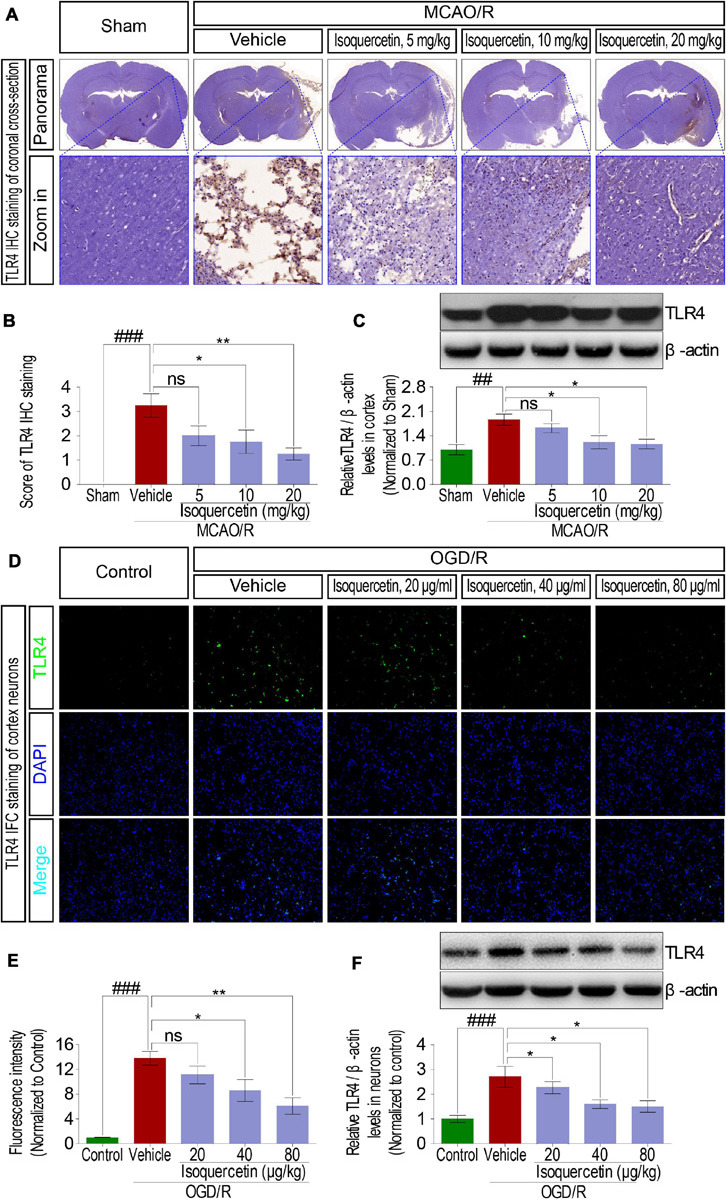
Effects of isoquercetin on the expression of TLR4 in the parietal cortex of rats after MCAO/R and primary culture of cortical neurons after OGD/R injury. **(A)** Representative micrographs of IHC staining of TLR4 in the parietal cortex of rats after MCAO/R and isoquercetin treatment. The zoom-in micrographs of equivalent regions in the parietal cortex were displayed. **(B)** Assessment analyses of IHC score of TLR4 in the parietal cortex of sham-operated, vehicle-treated, and isoquercetin-treated rats (*n* = 4). **(C)** The protein level of TLR4 in the parietal cortex of rats was assayed by western blot after MCAO/R injury and isoquercetin treatments (*n* = 3). **(D)** Representative fluorescence micrographs of TLR4 immunofluorescence staining of neurons after OGD/R and isoquercetin treatments. **(E)** Fluorescence intensity of TLR4 in neurons after OGD/R and isoquercetin treatment (*n* = 4). **(F)** The protein level of TLR4 in neurons was assayed by western blot analysis after OGD/R injury and isoquercetin treatments. Results were expressed as mean ± SEM. ^##^*p* < 0.01 and ^###^*p* < 0.001 vs. sham group or control group; *p < 0.05 and **p < 0.01 vs. vehicle-treated group.

In MCAO/R-exposed rats, inflammatory cytokines such as TNF-α, IL-1β, and IL-6 were verified to release, suggesting inflammatory response activation. Innate immune factor TLR4 is commonly deemed to participate in the inflammatory response process. Therefore, we analyzed the effect of isoquercetin on the expression level of TLR4 in MCAO/R-induced rats and OGD/R-exposed neurons. Protein expression level of TLR4 in the parietal cortex was significantly elevated in MCAO/R-induced rats shown by IHC (Figures 6A,B) and western blotting assays (Figure 6C). Isoquercetin treatment at 10 and 20 mg/kg elicited a significant decrease in TLR4 protein expressions as compared with vehicle-treated rats. Moreover, TLR4 expression was also elevated after OGD/R injury in primary culture of cortical neurons indicated by immunofluorescence chemistry (Figures 6D,E) and western blotting assays (Figure 6F). These elevated expressions of TLR4 in OGD/R neurons were also significantly inhibited by isoquercetin treatment at 40 and 80 μg/ml.

### Isoquercetin Exerted Neuroprotective Effects by Inhibiting C5aR1 Signaling

**FIGURE 7:**
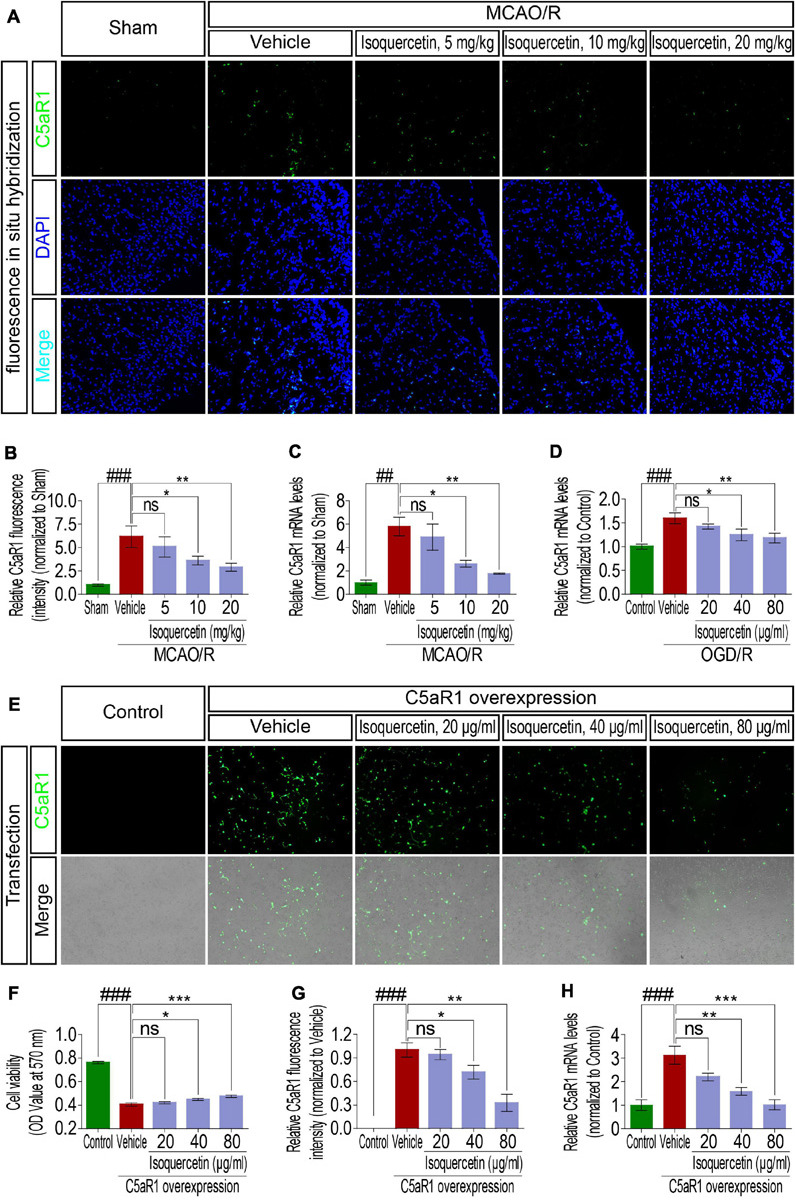
Effects of isoquercetin on C5aR1 expression in the parietal cortex of rats after MCAO/R and primary culture of cortical neurons after OGD/R injury and C5aR1 overexpression. **(A)** Representative micrographs of FISH of C5aR1 mRNA expression in the parietal cortex of sham-operated, vehicle-treated, and isoquercetin-treated rats. **(B)** The fluorescent intensity statistics of C5aR1 mRNA in the parietal cortex of sham-operated, vehicle-treated, and isoquercetin-treated rats (*n* = 4). **(C)** qPCR assay of C5aR1 mRNA expression levels in the parietal cortex of sham-operated, vehicle-treated, and isoquercetin-treated rats (*n* = 3). **(D)** qPCR assay of C5aR1 mRNA expression levels in control-, vehicle-, and isoquercetin-treated primary culture of cortical neurons (*n* = 3). **(E)** Representative micrographs of C5aR1 expression in primary culture of cortical neurons after over-expression and isoquercetin treatments. **(F)** Cell viability of cortical neurons after over-expression and isoquercetin treatments (*n* = 8). **(G)** The relative fluorescent intensity statistics of C5aR1 mRNA in cortical neurons after over-expression and isoquercetin treatments (*n* = 3). **(H)** qPCR assay of C5aR1 mRNA expression levels of cortical neurons after over-expression and isoquercetin treatments (*n* = 3). Results were expressed as mean ± SEM. ^##^*p* < 0.01 and ^###^*p* < 0.001 vs. sham or control group; *p < 0.05, **p < 0.01, and ***p < 0.001 vs. vehicle-treated group.

It has been demonstrated that C5aR1 participated in the pathological mechanism of brain injury after ischemic stroke. Therefore, in this study, we investigated the expression of C5aR1 by fluorescence *in situ* hybridization (FISH) and qPCR in the parietal cortex of rats after MCAO/R injury and isoquercetin treatment. The results presented in Figures 7A–C showed that MCAO/R injury enhanced C5aR1 expression level, which was significantly down-regulated by isoquercetin treatment at 10 and 20 mg/kg. Similar results were obtained under treatment with isoquercetin in OGD/R-exposed neurons (Figure 7D), in that isoquercetin at 40 and 80 μg/ml significantly decreased C5aR1 mRNA expression levels. In addition, the effects of isoquercetin on C5aR1 expression were determined in primary culture of cortical neurons after C5aR1 overexpression (Figures 7E–H). Transfection of C5aR1 mRNA labeled with fluorescent group significantly depressed the viability of cortical neurons. Also, isoquercetin treatment in C5aR1-overexpressed cortical neurons resulted in significant enhancements of cell viability and reductions of C5aR1 mRNA fluorescent intensity and C5aR1 mRNA expression level.

### cAMP-PKA and I-κB/NF-κB Signaling Were Involved in Neuroprotective Effects of Isoquercetin

**FIGURE 8:**
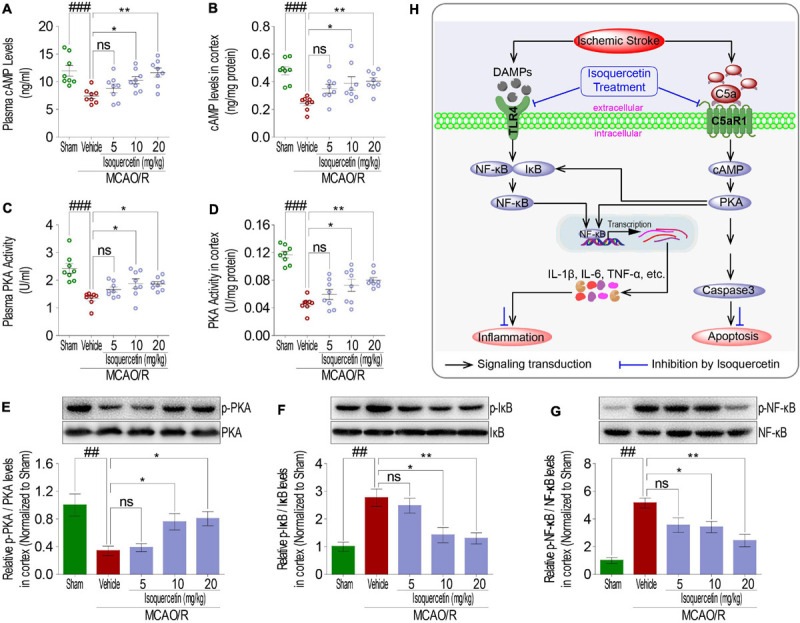
Effects of isoquercetin treatments on cAMP-PKA and I-κB/NF-κB signals in the parietal cortex of sham-operated, vehicle-treated, and isoquercetin-treated rats. **(A)** cAMP levels in plasma (*n* = 8). **(B)** cAMP levels in the parietal cortex (*n* = 8). **(C)** PKA activity in plasma (*n* = 8). **(D)** PKA activity in the parietal cortex (*n* = 8). The protein expression levels of PKA **(E)**, IκBα **(F)**, and NF-κB **(G)** in the parietal cortex of sham-operated, vehicle-treated, and isoquercetin-treated rats were determined by western blot (*n* = 3). **(H)** Isoquercetin participates in the regulation of neuron inflammation and apoptosis signal cascade in MCAO/R rats. Results were expressed as mean ± SEM. ^##^*p* < 0.01 and ^###^*p* < 0.001 vs. sham group; *p < 0.05 and **p < 0.01 vs. vehicle-treated group.

As membrane proteins, TLR4 and C5aR1 exert their receptor functions depending upon signaling transduction into intracellular cascading molecules. There has been lot of evidences that intracellular cAMP/PKA and I-κB/NF-κB signaling pathways participated in inflammatory response. Thus, cAMP, PKA activity, I-κB, and NF-κB were detected in rats after MCAO/R injury and isoquercetin treatment. MCAO/R injury induced activation of cAMP and PKA, characterized by the decreased cAMP levels (Figures 8A,B) and PKA activity (Figures 8C–E) in the plasma and cortex. Isoquercetin treatment at 10 and 20 mg/kg significantly enhanced the activation of cAMP and PKA in ischemic rats. Moreover, isoquercetin treatment at 10 and 20 mg/kg significantly reduced the phosphorylation of NF-κB and I-κB that resulted from MCAO/R injury (Figures 8F,G). These results demonstrated that isoquercetin protected against ischemic brain injury likely by modulating cAMP/PKA and I-κB/NF-κB cascade (Figure 8H).

## Discussion

We previously demonstrated that isoquercetin protected the hippocampal neuron against ischemia injury in rats (Wang et al., 2017). After the treatment, isoquercetin inhibited pro-inflammatory cytokine production in the hippocampus of MCAO/R rats by mediating TLR4 signaling. Consistent with our previous study, typical outcomes such as neurological deficits, cerebral edema and infarct, inflammatory cytokine release, cell damage, and apoptosis were apparently observed in ischemic brain of MCAO/R-induced rats, and OGD/R exposure elicited neuron death in *in vitro* model. Moreover, isoquercetin treatment obviously improved the animals’ survival rate and abovementioned outcomes in both MCAO/R-induced rats and OGD/R-exposed cortical neurons. Thus, we further investigated the molecular mechanism underlying these phenomena. C5aR1 can crosstalk with TLR4, which enhances the NF-κB-triggered cellular inflammation activation (Zaal et al., 2017a). Once excessively generated, C5aR1 plays an important role in the induction of inflammatory cytokines through downstream signal transduction cascades involving cAMP and PKA (Kim and Jang, 2017). Although, the mechanism through which C5aR1 conferred its regulation to modulate host functions remained elusive. In murine models that mimic some of immune-mediated diseases, treatment with the C5aR1 antagonist PMX53 improved disease outcome (Woodruff et al., 2003). In order to extend the scope of previous findings and gain further insight into the action of isoquercetin, we conducted this study.

In the present study, significant elevations of TLR4 and C5aR1 expression levels were identified in MCAO/R-injured rats, and the resultant inhibition of cAMP/PKA cascade contributed to the regulatory pathways of the activation of NF-κB through regulation of phosphorylation of IκBα. Then, the inflammatory cytokines TNF-α, IL-1β, and IL-6’s expression process was significantly enhanced. In addition, IκBα has also been reported to be the potential substrate of PKA (Chang et al., 2017). That is, TLR4 and C5aR1 represent an alternative upstream regulator in activating NF-κB by inhibiting cAMP/PKA signals and participate in TNF-α, IL-1β, and IL-6 production in MCAO/R-induced brain injury. Apart from TLR4 to C5aR1 blockade, isoquercetin treatment elevated cAMP/PKA activation and showed a suppressive effect on NF-κB and IκBα phosphorylation, accompanied by the decreased expression levels of TNF-α, IL-6, and IL-1β in the ischemic parietal cortex of SD rats. In addition, Caspase 3, a major executor of cell apoptosis (Jani et al., 2004), was found to be significantly increased in the parietal cortex of MCAO/R-exposed rats. Activated cAMP/PKA signaling has been demonstrated to inhibit apoptosis by suppressing the activation of Caspases 3 (Li et al., 2000). Consistent with these reports, MCAO/R resulted in reduced cAMP/PKA signals and activated Caspase 3 expression in the parietal cortex of SD rats. Interestingly, these effects were improved by isoquercetin treatment in the parietal cortex of MCAO/R-induced rats. Combined with the results from TUNEL staining in the parietal cortex of ischemic rats, isoquercetin treatment robustly ameliorated cell apoptosis against MCAO/R injury. Collectively, cAMP/PKA, IκB/NF-κB, and Caspase 3 signaling pathways mediated by TLR4 and C5aR1 were identified to be essential for the inflammatory response and cell apoptosis in ischemic stroke of MCAO/R-exposed rats. Moreover, isoquercetin remarkably alleviated neurological deficits, cerebral edema and infarct, inflammatory cytokine release, cell damage, and apoptosis in MCAO/R-treated rats, possibly by enhancing cAMP/PKA, inhibiting IκB/NF-κB, and Caspase 3 signaling pathway through TLR4 and C5aR1 down-regulations.

In this study, I/R-induced brain injury indicated by inflammatory responses and cell apoptosis is testified to conjunct with C5aR1 and TLR4 mediating pathway, which involves cAMP/PKA IκB/NF-κB, and Caspase 3 signaling transduction. Through these signals, isoquercetin displayed potential neuroprotective effects that neurological deficits, cortical edema, cell damage, inflammatory cytokine release, cell apoptosis, and etc. were improved significantly. Therefore, we conclude that targeting C5aR1- and TLR4-involved signaling pathway is efficacious to treat I/R-induced brain injury in rats with the neuroprotective agent isoquercetin as shown in Figure 8H. Here, we provide a possible therapeutic strategy and a potential neuroprotective agent in ischemic stroke.

## Data Availability Statement

The raw data supporting the conclusions of this article will be made available by the authors, without undue reservation, to any qualified researcher.

## Ethics Statement

The animal study was reviewed and approved by the Lab Animal Ethical Committee of Nantong University.

## Author Contributions

YS and CW designed the experiments, analyzed the data, and prepared the manuscript. JL, XC, XF, YJ, JG, and JL did the experiments. XL contributed in the conception and design of the manuscript. All authors contributed to the article and approved the submitted version.

## Conflict of Interest

The authors declare that the research was conducted in the absence of any commercial or financial relationships that could be construed as a potential conflict of interest.
