# Supplementary material for: Isoquercetin Improves Inflammatory Response in Rats Following Ischemic Stroke
Source: Front Neurosci. 2021 Feb 9;15:555543. doi: 10.3389/fnins.2021.555543 (PMC7900503; doi:10.3389/fnins.2021.555543)
Supplement: Supplementary file 1 [file Table_1.DOCX]

**Supplementary Materials**

**Title: Isoquercetin improves inflammatory response in rats following ischemic stroke**

Yunwei Shi ^1, 5^, Jiaxing Liu ^1, 5^, Xinyi Chen ^1, 5^, Xingjuan Fan ^2^, Ying Jin ^1^, Jingxiao Gu ^3^, Jiale Liang ^3^, Xinmiao Liang ^4, 6^, Caiping Wang ^1, 6^

^1^ *Key Laboratory of Neuroregeneration of Jiangsu and Ministry of Education, Co-innovation Center of Neuroregeneration, Nantong University, Nantong 226001, Jiangsu, China;*

^2^ *Department of Neurology, Affiliated Hospital of Nantong University, Nantong 226001, Jiangsu, China;*

^3^ *Medical school, Nantong University, Nantong 226001, Jiangsu, China;*

^4^ *Dalian Institute of Chemical Physics, Chinese Academy of Sciences, Dalian 116023, Liaoning, China;*

^5^ These authors contributed equally to this work;

^6^ Authors for correspondence:

Caiping Wang, Ph. D

Key Laboratory of Neuroregeneration of Jiangsu and Ministry of Education, Nantong University.

Qixiu Road 19, Nantong 226001, Jiangsu, China.

Email: cai-pingwang@163.com; caipingwang@ntu.edu.cn

Xinmiao Liang, Ph. D

Dalian Institute of Chemical Physics, Chinese Academy of Sciences.

Email: liangxm@dicp.ac.cn

**Supplementary Data** **Legends**

**Table S1.** Antibodies used for western blot, immunohistochemistry and immunofluorescence analyses.

**Figure S1** The effects of isoquercetin at different doses (0 - 320 μg/ml) for 24 h on the primary cultured cortical neurons of SD rats. A, The cell index was recorded at 25-min intervals by RTCA-MP xCELLigence system. B, Cell index analyses at 5 time points. Results were expressed as mean ± SEM. **p* < 0.05 versus normal group.

**Supplementary Movies**

The movies of cell morphological changes in primary cultured cortical neurons of SD rats after OGD/R and isoquercetin treatments by time-lapse imaging. Movie 1, Control group. Movie 2, OGD/R group. Movie 3, OGD/R and 20 μg/ml isoquercetin-treated group. Movie 4, OGD/R and 40 μg/ml isoquercetin-treated group. Movie 5, OGD/R and 80 μg/ml isoquercetin-treated group.
