# Supplementary material for: Isoquercetin Improves Inflammatory Response in Rats Following Ischemic Stroke
Source: Front Neurosci. 2021 Feb 9;15:555543. doi: 10.3389/fnins.2021.555543 (PMC7900503; doi:10.3389/fnins.2021.555543)
Supplement: Supplementary file 2 [file Table_2.DOC]

**Table S1.** Antibodies used for Western blot and immunohistochemistry and immunofluorescence analyses.

Company Description Catalog number

Cell Signaling Technology, Inc. Phospho-NF-κB p65 (Ser536) (93H1) Rabbit mAb #3033S

(Boston, MA, USA) Phospho-IκB (Ser32/36) (5A5) Mouse mAb #9246S

NF-κB p65 (D14E12) XP® Rabbit mAb #8242

Abcam (China, Mainland), Ltd. Rabbit monoclonal IκB antibody ab32518

Rabbit polyclonal IL6 antibody ab6672

Rabbit polyclonal IL1 beta antibody ab9722

Anti-TNF alpha antibody ab6671

Anti-PKA alpha/beta/gamma (catalytic subunit) (phosphoT197) antibody ab75991

Anti-PKA alpha + beta (catalytic subunits) antibody ab216572

Novus Bio-Techne China Co. Ltd.  Monoclonal TLR4 Antibody (76B357.1) NB100-56566

Proteintech Group, Inc. TLR4 Rabbit Polyclonal antibody 19811-1-AP

(Wu Han, SanYing) Rabbit polyclonal Caspase 3/p17 antibody 19677-1-AP

Beta Actin Mouse Monoclonal antibody 60008-1-Ig

Mouse monoclonal GAPDH antibody 60004-1-Ig

HRP-goat anti-rabbit IgG (H + L) 00001-1

HRP-goat anti-mouse IgG (H + L) 00001-2

HRP, horseradish peroxidase; IgG, immunoglobulin G.
