# Supplementary figures and images for: Isoquercetin Improves Inflammatory Response in Rats Following Ischemic Stroke
Source: Front Neurosci. 2021 Feb 9;15:555543. doi: 10.3389/fnins.2021.555543 (PMC7900503; doi:10.3389/fnins.2021.555543)

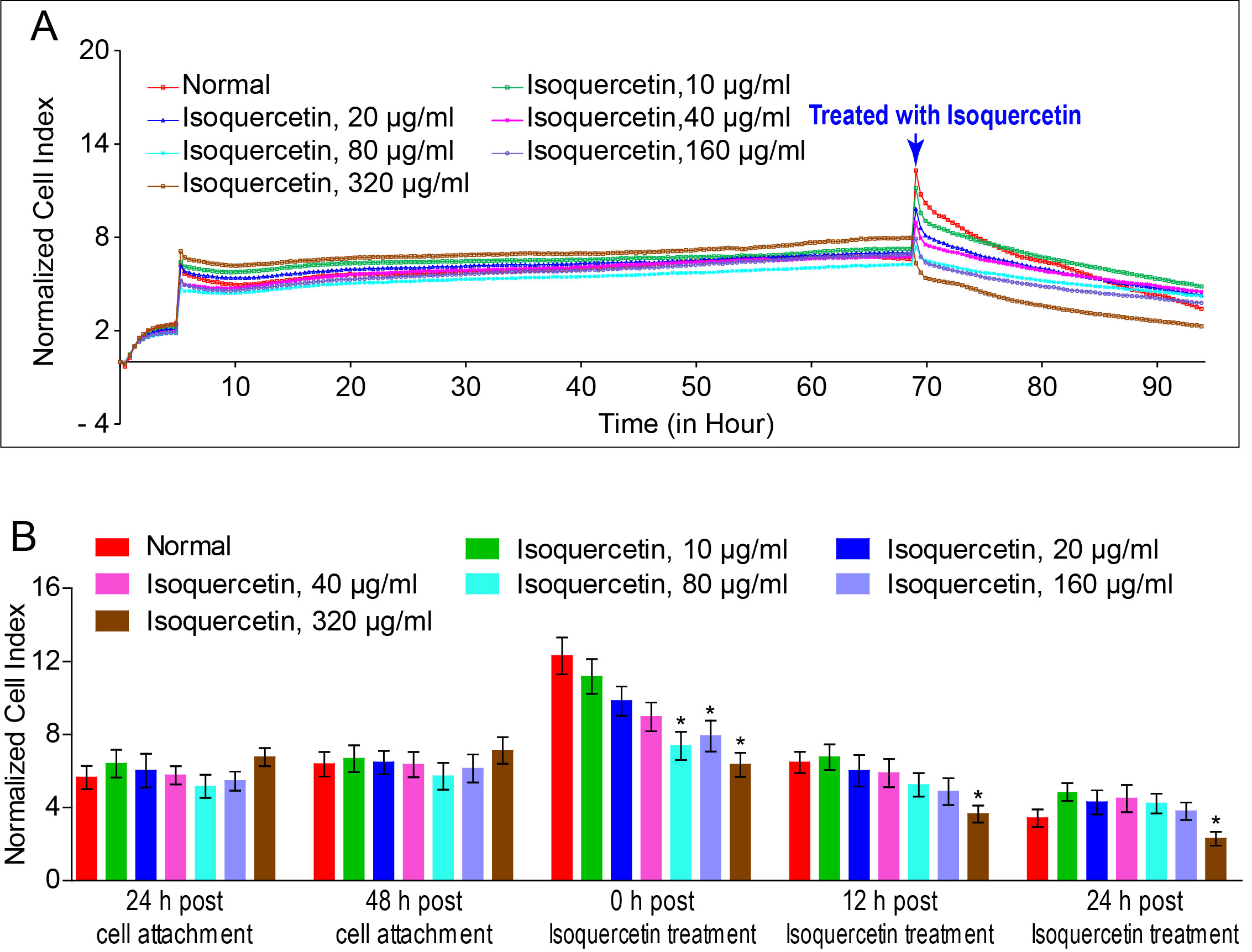

Supplement: Supplementary file 8 [file Image_1.JPEG]

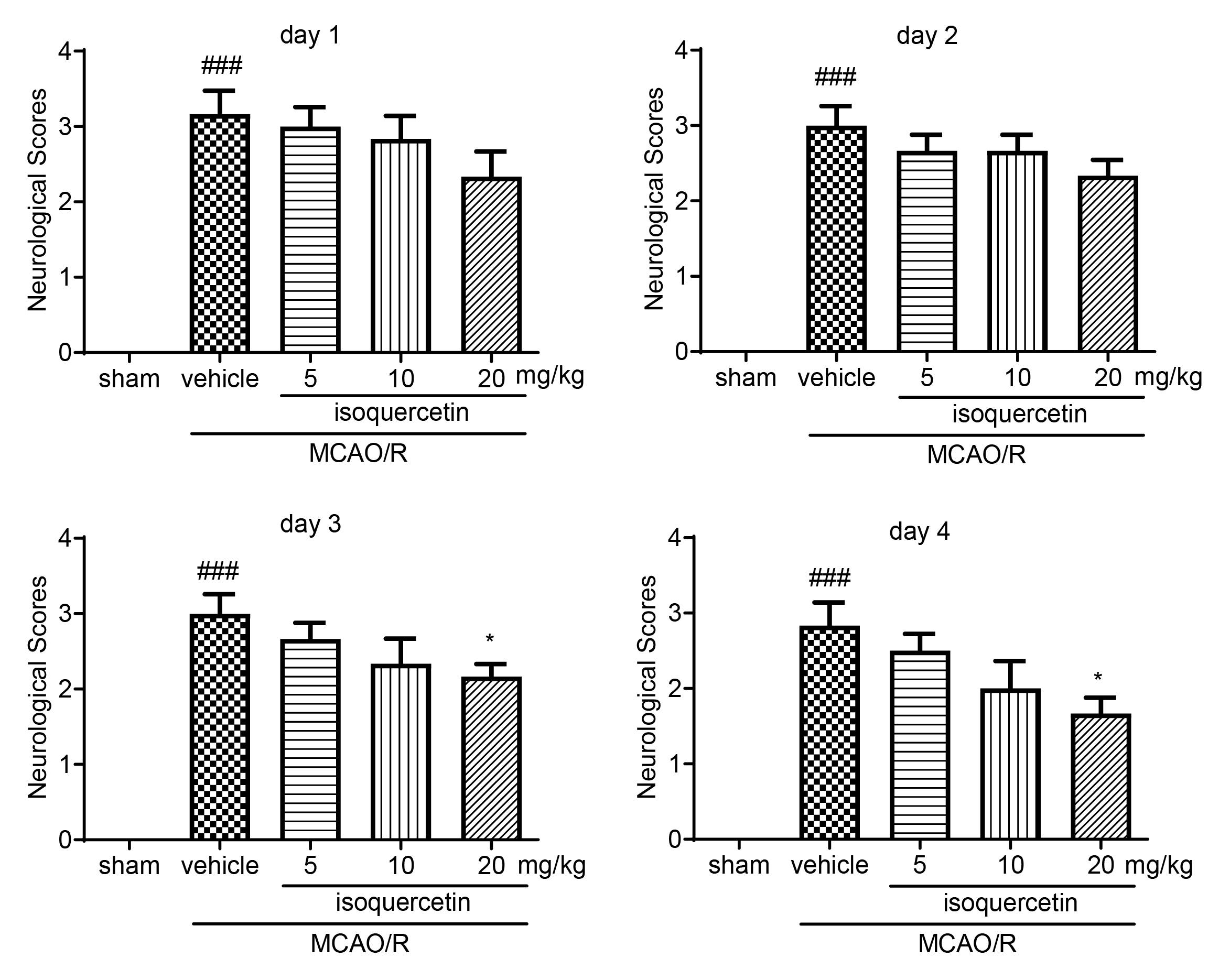

Supplement: Supplementary file 9 [file Image_2.JPEG]

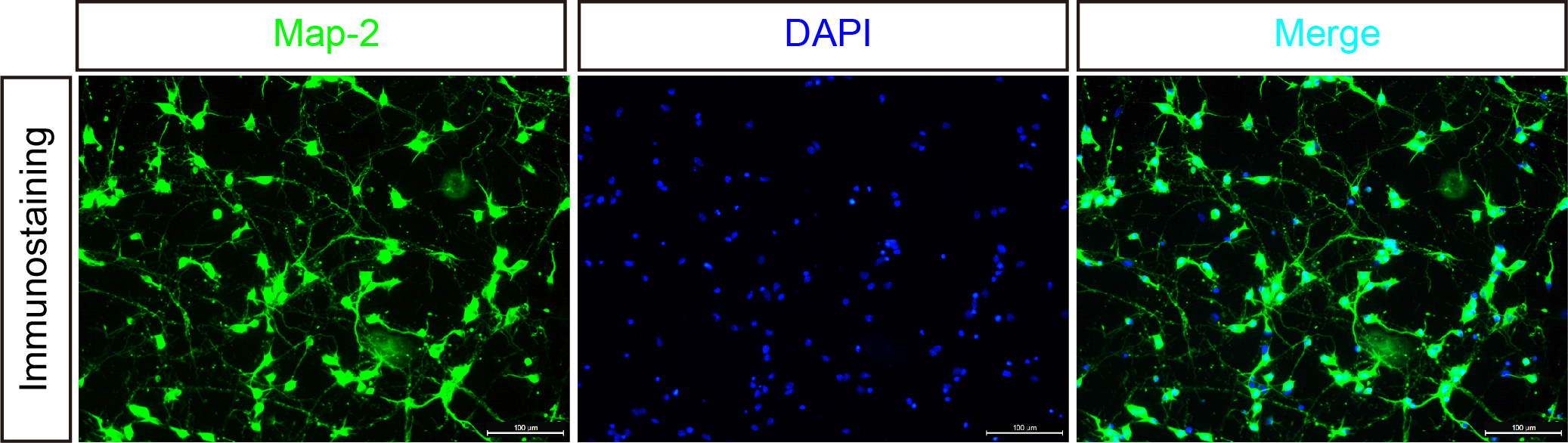

Supplement: Supplementary file 10 [file Image_3.JPEG]

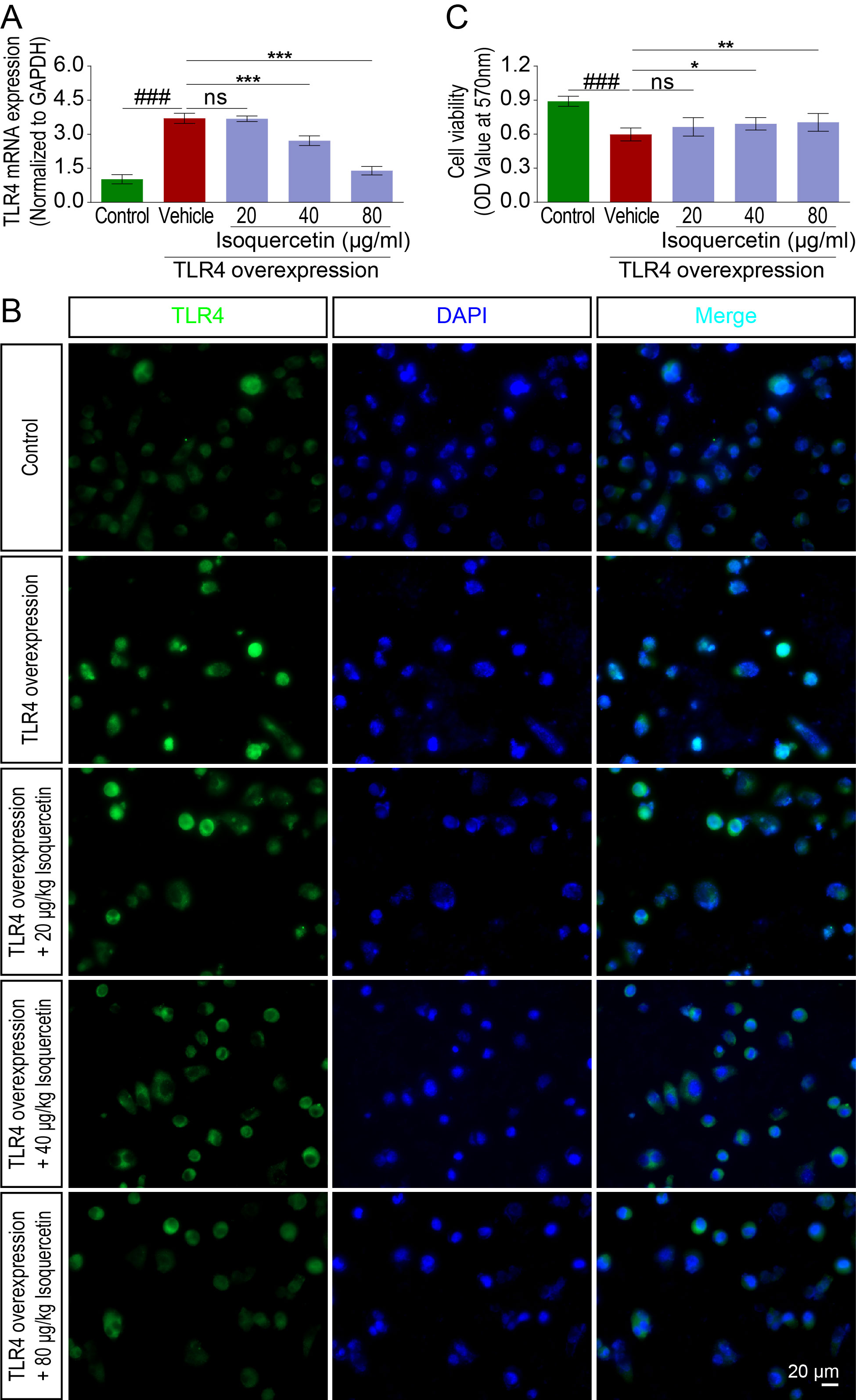

Supplement: Supplementary file 11 [file Image_4.JPEG]

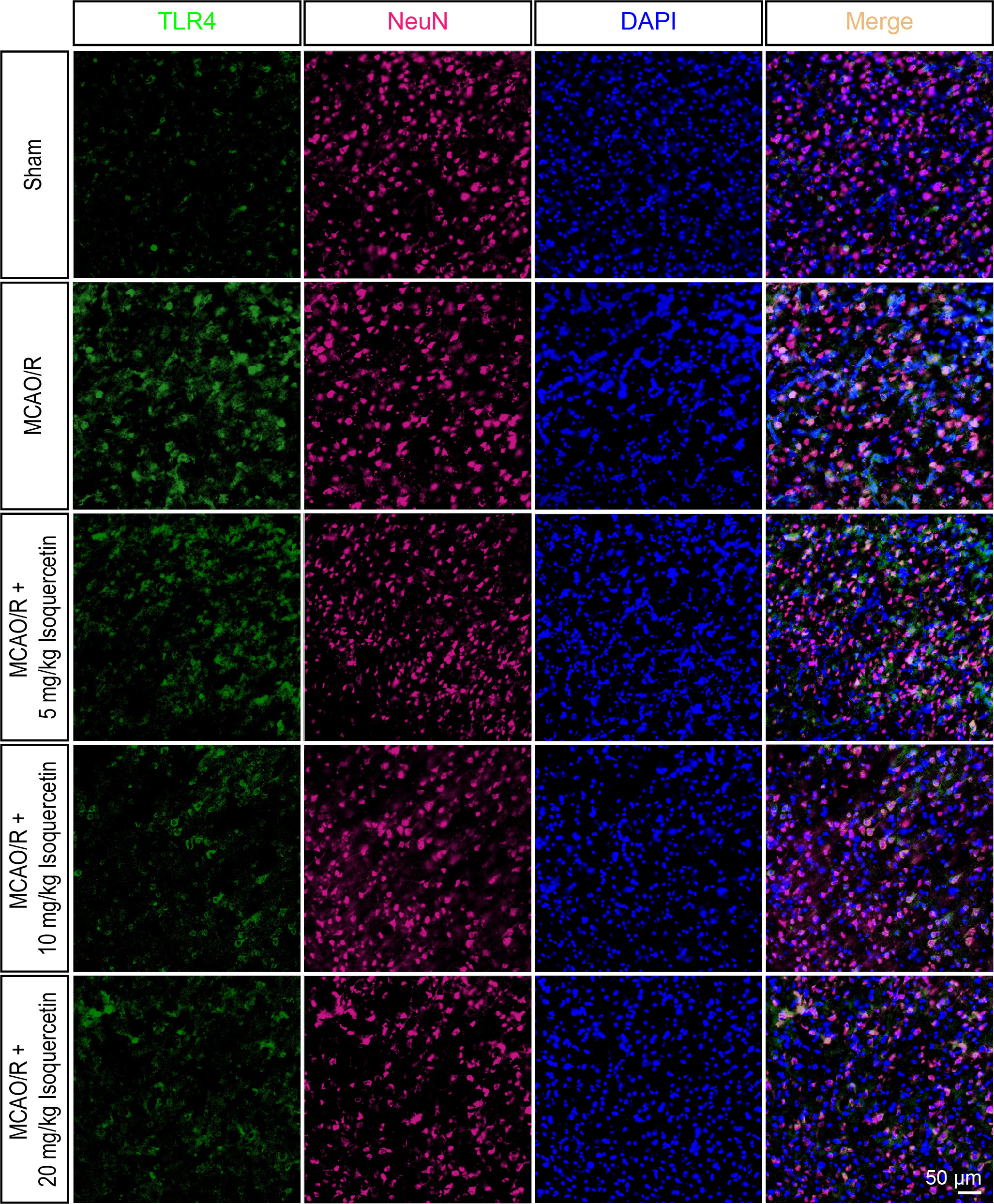

Supplement: Supplementary file 12 [file Image_5.JPEG]

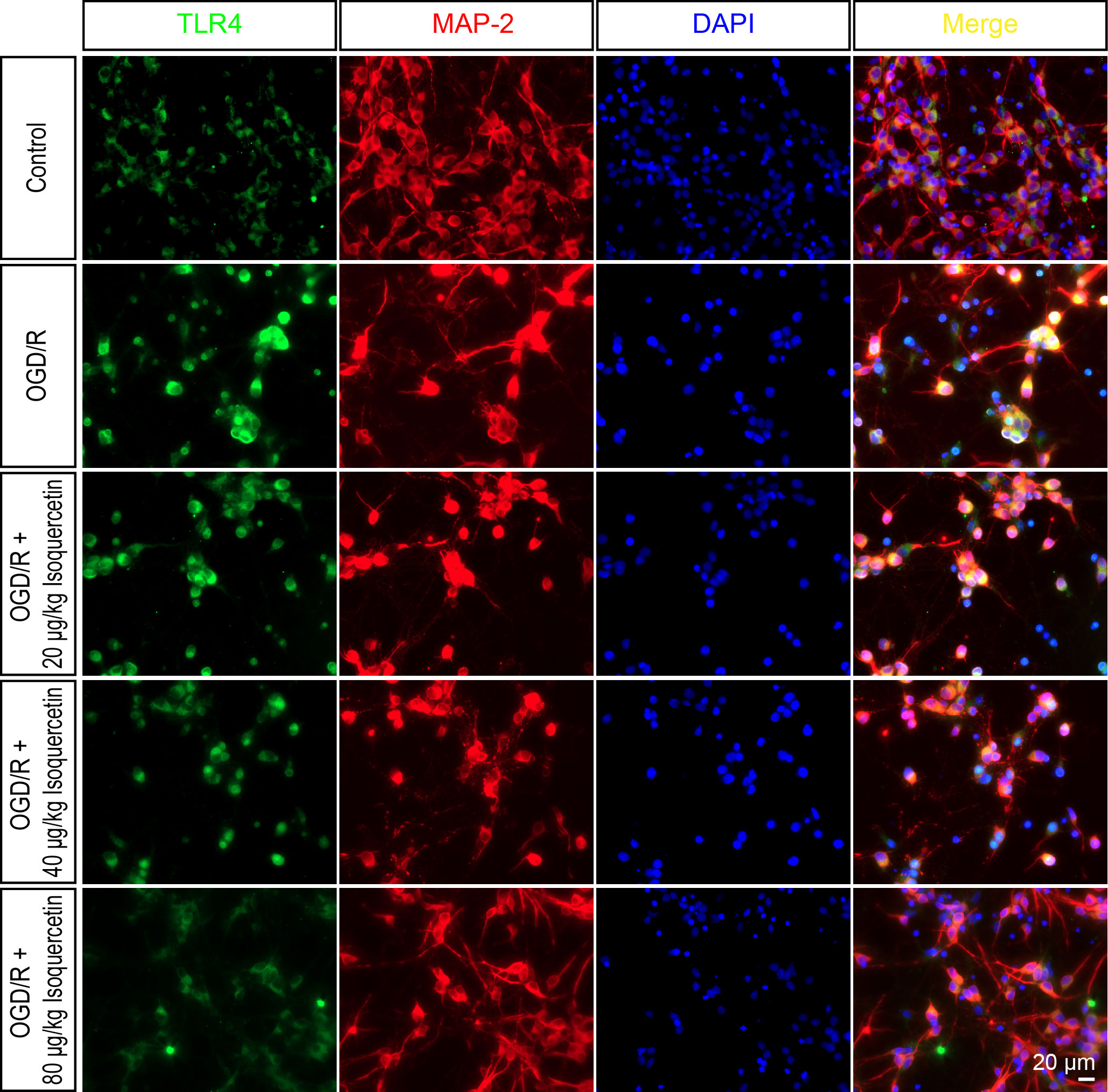

Supplement: Supplementary file 13 [file Image_6.JPEG]
